# Sedentary behavior, physical activity and cardiorespiratory fitness on leukocyte telomere length

**DOI:** 10.15171/hpp.2017.05

**Published:** 2016-12-18

**Authors:** Meghan K. Edwards, Paul D. Loprinzi

**Affiliations:** ^1^Physical Activity Epidemiology Laboratory, Department of Health, Exercise Science and Recreation Management, The University of Mississippi, University, MS 38677, USA; ^2^Jackson Heart Study Vanguard Center of Oxford, Center for Health Behavior Research, Physical Activity Epidemiology Laboratory, Department of Health, Exercise Science and Recreation Management, The University of Mississippi, University, MS 38677, USA

**Keywords:** Aging, Chronic disease, Epidemiology, Exercise

## Abstract

**Background:** Emerging work is starting to investigate the cumulative effects of moderate-to-vigorous physical activity (MVPA), sedentary behavior and cardiorespiratory fitness on health. The objective of this study was to examine the cumulative and independent associations of MVPA, sedentary behavior and cardiorespiratory fitness on leukocyte telomere length (LTL).

**Methods:** Data from the 1999-2002 National Health and Nutrition Examination Survey (NHANES) were used (N = 1868 adults 20+ years); analyzed in 2016. Sedentary behavior and MVPA were subjectively assessed with cardiorespiratory fitness determined from a submaximal treadmill-based test; participants were classified as above or below the median values for each of these three parameters. A blood sample was obtained from each participant to assess LTL via quantitative polymerase chain reaction, with participants grouped into LTL tertiles.

**Results:** Participants who engaged in higher MVPA, sat less and had higher cardiorespiratory fitness had an increased odds (ranging from 85% to 105%) of being in LTL tertile 3 (vs. 1). In an extended adjusted multinomial logistic regression model, only MVPA was positively associated with LTL (odds ration [OR] = 1.37; 95% CI: 0.99-1.90; P = 0.05).

**Conclusion:** All three behavior characteristics, but particularly MVPA, may be important in preserving LTLs.

## Introduction

Telomeres are nucleoprotein structures that promote stability of chromosomes by capping their ends.^1^ Given their prominent role in maintaining chromosomal health, telomere dysfunction can have deleterious consequences.^2^ Notably, cellular senescence has been shown to influence both degenerative and hyperplastic diseases associated with aging.^3^ Research on a senescence-associated secretory phenotype (SASP), a feature of many senescent cells, has helped to foster a better understanding of the impact of telomeres on aging and age-related pathology.^3^ A prominent conclusion drawn from the study of the SASP is that a variety of SASP proteins (e.g., interleukin-6, granulocyte-macrophage colony stimulating factor, monocyte chematactic protein-2, specific insulin-like growth factor-binding proteins), induced at the level of mRNA,^4^ are responsible for promoting chronic inflammation.^4^ This is important, as chronic systemic inflammation has been directly associated with a variety of age-related diseases (e.g., Alzheimer disease, type 2 diabetes, cancer, atherosclerosis, etc.).^5^

Ultimately, the body of research on telomeres has demonstrated that shortened leukocyte telomere length (LTL) is a hallmark characteristic of aging^6^ and is associated with a number of chronic diseases (e.g., cardiovascular disease, cancer, dementia, mortality).^7^ While often regarded as a contributing factor to age-related processes, shortened or otherwise dysfunctional telomeres have also been shown to have influences throughout the lifespan (e.g. promotion of genomic instability).^2^ Thus, a better understanding of the variables (especially modifiable health behaviors) that influence telomere length may be critical for the prevention or attenuation of a number of diseases.

Of notable interest to the topic of this study, the well-established health benefits of physical activity (e.g. reduced risk for chronic diseases such as cardiovascular disease, type 2 diabetes, and certain types of cancer)^8^ have been demonstrated to extend to LTL (i.e., a positive association between physical activity and LTL has been shown).^9^ Cardiorespiratory fitness has also been demonstrated to have an independent positive association with LTL.^10^ Alternatively, and consistent with other maladaptive health outcomes associated with sedentary behavior (e.g., increased risk for obesity, type 2 diabetes and mortality),^11^ there is evidence for an independent negative association between sedentary behavior and LTL.^12^

In addition to evaluating single-variable associations with health, recent research has focused on the simultaneous evaluation of multiple factors in order to determine their unique, independent associations, as well as any possible combined associations with a given health outcome.^13,14^ Studying both independent and combined associations between different variables and health outcomes may be a strategic, important method to increase our understanding of complex health-related processes. As mentioned previously, independent associations between physical activity, sedentary behavior, and cardiorespiratory fitness with LTL have been established.^9,10,12,15,16^ An investigation of their potential combined associations on LTL, however, has not yet been conducted. This is important to evaluate, because as previously noted, shortened LTL is associated with various chronic diseases.^7^ Thus, the purpose of this study was to evaluate both the independent and combined associations of physical activity, sedentary behavior, and cardiorespiratory fitness with LTL.

## Materials and Methods

### Design and participants

Data were extracted from the 1999-2002 National Health and Nutrition Examination Survey (NHANES); these are the only current NHANES cycles with telomere length data, and notably, these cycles do not contain objectively-measured physical activity/sedentary data (subjective measures described below). Analyses are based on data from 1868 adults (20-49 years; age range among adults eligible for the NHANES cardiorespiratory fitness test) who provided complete data for the study variables. The NHANES is an ongoing survey conducted by the Centers for Disease Control and Prevention that uses a representative sample of non-institutionalized US civilians selected by a complex, multistage, stratified, clustered probability design. Details have been described elsewhere.^17^ Briefly, the multistage design consists of 4 stages, including the identification of counties, segments (city blocks), random selection of households within the segments, and random selection of individuals within the households. Further information on NHANES methodology and data collection is available on the NHANES website (http://www.cdc.gov/nchs/nhanes.htm).

### Leukocyte telomere length 

Detailed methodology of the NHANES procedures for assessing LTL has been previously reported.^18^ Briefly, DNA was extracted from whole blood with the LTL assay performed using quantitative polymerase chain reaction to measure LTL relative to standard reference DNA (T/S ratio).^18^ Each sample was assayed at least twice, and among samples with a T/S ratio within 7% variability, the average value was used; for samples with a variability of greater than 7%, a third assay was run and in this case, the average of the two closest T/S values was used.

### Measurement of screen-based sedentary behavior

Participants were asked, “*Over the past 30 days, on a typical day how much time altogether did you spend sitting and watching TV or videos or using a computer outside of work*?” Response options were none, less than 1 hour, 1 hour, 2 hours, 3 hours, 4 hours, and 5 or more hrs. This screen-based sedentary behavior item has demonstrated some evidence of convergent validity by correlating with body mass index categories.^19^ Using data from the 2003-2006 NHANES (cycles with objective ‘overall’ sedentary data), the author computed the correlation between this self-report screen-based sedentary behavior item and identical categories (h/d) of accelerometer-determined sedentary behavior (counts/min <100); a weak statistically significant association (r = 0.10, *P* < 0.0001) was observed, which is not unexpected as this self-report screen-based sedentary item only assessed non-occupational sedentary behavior, whereas accelerometry assesses overall daily sedentary behavior. This observed correlation is within the range (r = -0.19 to 0.80) of a review paper documenting the concurrent validity of television viewing time and other non-occupational sedentary behaviors (referent measures included heart rate monitoring, behavioral logs and accelerometry combined with behavioral logs)^20^; notably, only 3 of the evaluated studies from this review examined the validity of self-reported television viewing time and other non-occupational sedentary behaviors. This review, did, however, demonstrate moderate-to-high reliability of these measures (the majority of the ICC’s were > 0.5).

### Measurement of physical activity

As discussed elsewhere,^21,22^ participants were asked open-ended questions about participation in leisure-time physical activity over the past 30 days. Data was coded into 48 activities, including 16 sports-related activities, 14 exercise-related activities, and 18 recreational-related activities.

For each of the 48 activities where participants reported moderate or vigorous-intensity for the respective activity, they were asked to report the number of times they engaged in that activity over the past 30 days and the average duration they engaged in that activity.

For each of activity, metabolic equivalent of task (MET)-min-month was calculated by multiplying the number of days, by the mean duration, by the respective MET level (MET-min-month = days*duration*MET level). The MET levels for each activity are provided elsewhere.^23^ Notably, a value of 2000 MET-min-month is consistent with government physical activity guidelines.^24^ The specific physical activity assessment used herein has demonstrated evidence of convergent validity by correlating with accelerometer-assessed physical activity.^21,22^

### Measurement of Cardiorespiratory Fitness (i.e., maximum oxygen consumption [VO_2max_])

At the mobile examination survey, participants aged 12-49 years old (only those 20-49 evaluated herein) were eligible for the treadmill-based cardiorespiratory fitness component. Participants were excluded from this component based on certain medical conditions (e.g., previously diagnosed with coronary heart disease or self-reported heart problems), medications (e.g., lidocaine), physical limitations (e.g., difficulty walking up ten steps without resting), limits on resting heart rate (i.e., > 100 beats per minute) and blood pressure (systolic blood pressure > 180 mm Hg; diastolic blood pressure > 100 mm Hg), irregular resting heart rates (> 3 beats per minute), and other reasons specified by the participant or MEC physician or staff (e.g., hospitalized in the previous 3 months).

The treadmill-based cardiorespiratory fitness exam was performed by trained health technicians. The protocol employed was a submaximal treadmill test. Participants were assigned to one of eight treadmill test protocols. Determining which treadmill test protocol to use was based on the participant’s predicted VO_2max_ using the Jackson et al^25^ prediction equation. The objective of each protocol was to elicit a heart rate that was approximately 75% of the participant’s age-predicted maximum heart rate (i.e., 220-age) by the conclusion of the test. Each treadmill protocol included a 2-minute warm-up period, two 3-minute exercise stages, and a 2-minute cool-down period. During the treadmill test, heart rate was monitored continuously using an automated monitor with four electrodes connected to the thorax and abdomen of the participant and was recorded at the end of the warm-up period, each exercise stage, and each minute of recovery. Because the relationship between heart rate and oxygen consumption is assumed to be linear during exercise,^26^ VO_2max_ was estimated by measuring the heart rate response to known levels of submaximal work. VO_2max_ testing has long-since served as a standard of oxygen consumption,^27^ to which numerous predictive submaximal tests have been compared to.^28^ Submaximal testing procedures have been developed and utilized in a variety of settings (e.g., clinical screening for cardiovascular-related diseases), due to the contraindications of maximal testing among individuals with restrictive cardiopulmonary, musculoskeletal and neuromuscular impairments. Notably, predictive submaximal treadmill testing protocols generally report adequate levels of criterion validity when compared to VO2max testing.^28^ For instance, the modified Bruce treadmill test,^29^ single stage submaximal treadmill walking test,^30^ and the 12-minute run test^31^ have each demonstrated Pearson correlation coefficients (when compared to maximal treadmill testing) between 0.86-0.98.

### Calculation of physical activity, cardiorespiratory and sedentary behavior index score

A PACS (Physical Activity Cardiorespiratory Sedentary) score was created that ranged from 0-3, indicating the number of positive characteristics. For each variable, a score of 0/1 was created using the median split method. The median split method was specifically employed to ensure an adequate cell size for rendering of reliable point estimates. Thus, those above the sample MVPA median of 1,835 MET-min-month were given a score of “1”; those below the sample sedentary median of 2 h/d were given a score of “1”; and those above the sample VO_2max_ median of 39 mL/kg/min were given a score of “1.”

### Statistical Analyses

All analyses accounted for the complex survey design employed in NHANES. Statistical analyses were performed using procedures from survey data using Stata (v.12, College Station, Texas, USA). Polytomous regression was used to examine the odds of being in the lowest tertile^32,33^ (vs. upper tertiles) of LTL based on PACS; those with a PACS index score of “0” served as the referent group. Assumptions of the polytomous regression were checked and confirmed (e.g., dependent variable is categorical, independence of observations, no multicollinearity, no outliers).

Four models were computed, including (1) an unadjusted model, (2) age-adjusted model, (3) minimally adjusted model, and (4) extended adjusted model. In the minimally adjusted model, covariates included, age (years), gender, race-ethnicity (Mexican American, other Hispanic, non-Hispanic white, non-Hispanic black, and other/multi-race) and measured body mass index (kg/m^2^). The extended adjusted model included the same covariates in the minimally adjusted model plus physician-diagnosed hypertension (yes/no), self-reported smoking status (current, former, never), and physician-diagnosed diabetes (yes/no). Statistical significance was set at *P *< 0.05.

## Results

Table 1 displays the unweighted characteristics of the study variables. Table 2 displays the results for the polytomous logistic regression examining the association between PACS index score and telomere length. For all models (unadjusted to extended adjusted), those with a PACS index of 3 (vs. 0) had an increased odds (ranging from 85% to 105%) of being in LTL tertile 3 (vs. 1). That is, those who engaged in less sedentary behavior, engaged in greater physical activity, and had higher cardiorespiratory fitness had the highest LTL. Additional analyses evaluated the potential independent associations of the individual PACS parameters. In an extended adjusted multinomial logistic regression model, only MVPA (odds ratio [OR] = 1.37; 95% CI: 0.99-1.90; *P* = 0.05), but not sedentary behavior (OR = 1.06-0.76-1.48; *P* = 0.68) or cardiorespiratory fitness (OR = 0.97; 95% CI: 0.71-1.32; *P* = 0.85), was associated with being in the upper (vs. lower) LTL. Further, there was no evidence of an interaction effect of PACS and age or PACS and gender, on LTL (data not shown).

**Table 1:** Unweighted characteristics of the study variables (N=1868)

**Variable**	**Mean/Proportion**	**Range**
Telomere length, mean T/S ratio	1.13	0.54-9.42
Age, years	33.7	20-49
Male, %	50.8	
Race-Ethnicity, %		
Mexican American	26.5	
Other Hispanic	5.3	
White	48.8	
Black	16.9	
Other/multi-race	2.5	
BMI, mean kg/m^2^	27.3	15.5-57.5
Hypertension, %	9.0	
Current smoker, %	25.7	
Diabetes, %	1.8	
PACS index, %		
0	19.3	
1	38.7	
2	31.1	
3	11.0	

Abbreviations: BMI, body mass index; PACS, Physical Activity Cardiorespiratory Sedentary

**Table 2:** Polytomous logistic regression examining the association between PACS index score and telomere length, 1999-2002 NHANES (N = 1868).

**PACS index**	**Odds ratio (95% CI)**		
	**Lowest LTL tertile**	**Middle LTL tertile**	**Upper LTL tertile**
Unadjusted			
1 vs. 0	Referent	0.92 (0.62-1.38)	0.99 (0.64-1.53)
2 vs. 0	Referent	0.73 (0.46-1.15)	1.06 (0.66-1.69)
3 vs. 0	Referent	1.64 (1.05-2.57)	1.96 (1.14-3.38)
Age-adjustment^a^			
1 vs. 0	Referent	0.93 (0.61-1.44)	1.01 (0.64-1.60)
2 vs. 0	Referent	0.73 (0.46-1.17)	1.06 (0.65-1.72)
3 vs. 0	Referent	1.69 (1.06-2.71)	2.05 (1.14-3.67)
Minimal adjustment^b^			
1 vs. 0	Referent	0.91 (0.59-1.41)	1.02 (0.64-1.63)
2 vs. 0	Referent	0.68 (0.43-1.06)	1.02 (0.61-1.69)
3 vs. 0	Referent	1.53 (1.01-2.33)	1.89 (1.05-3.40)
Extended adjustment^c^			
1 vs. 0	Referent	0.90 (0.58-1.39)	1.01 (0.64-1.59)
2 vs. 0	Referent	0.66 (0.42-1.04)	1.00 (0.60-1.64)
3 vs. 0	Referent	1.46 (0.94-2.26)	1.85 (1.04-3.27)

Abbreviations: LTL, leukocyte telomere length; PACS, Physical Activity Cardiorespiratory Sedentary. ^a^ Adjusted for age. ^b^ Adjusted for age, gender, race-ethnicity, and body mass index. ^c^ Adjusted for age, gender, race-ethnicity, body mass index, hypertension, smoking status and diabetes status.

## Discussion

Evaluating factors that influence telomere length is important, given that shorter LTL is not solely a marker of aging and may contribute to health consequences across the lifespan. Shortening of the DNA component of telomeres may induce genomic instability, cellular senescence, apoptosis, and as a result, may possibly facilitate cardiovascular disease risk.^7^ As a conclusive result of these consequences, studying factors that influence telomere length (as was the purpose of this study) could provide important insights that assist in identifying factors that may help to attenuate risks associated with these diseases. The main finding of the present study was that adults with all three behavioral characteristics (i.e., individuals who were physically active, engaged in less sedentary behavior and had higher levels of cardiorespiratory fitness) had the longest LTL. Notably, only MVPA was independently associated with LTL.

These “cumulative” findings align with previous work showing that these behavioral characteristics may play an important role in preserving the length of the LTL.^9,10,12,15,16^ These findings add to the current emerging body of literature on this topic suggesting that, perhaps, MVPA may play the most important role in telomere preservation. These findings are in alignment with a recent sedentary behavior meta-analysis by Biswas et al, which found that MVPA generally attenuated the negative effects of sedentary behavior on cardiovascular disease, diabetes, cancer, and all-cause mortality.^34^ Presumably, and as demonstrated in our “independent” analytic model, MVPA may have attenuated the effects of sedentary behavior in this study, resulting in a non-significant association between sedentary behavior and LTL. Of relatable interest, a recent study compared the associations of different modalities of physical activity (e.g., bicycling, walking, lifting weights) of varying metabolic equivalent units (METs),^23^ indicating that running was the only evaluated physical activity associated with LTL.^16^ This provides evidence that differing physical activity modes or intensities may have differential effects on LTL. This evidence was corroborated with our current study demonstrating that MVPA (METs ≥ 3.0)^35^ had a significant association with LTL, whereas sedentary behavior (1-1.5 METS)^36^ did not. While cardiorespiratory fitness may be a by-product of physical activity (i.e., physical activity has been shown to have significant, positive correlations with cardiorespiratory fitness; those with higher levels of physical activity tend to have higher levels of cardiorespiratory fitness),^37^ there are other factors that contribute to one’s cardiorespiratory fitness (e.g., maximal heritability estimates have previously been reported at approximately 50%),^38,39^ which may partially explain why we did not observe a significant association between cardiorespiratory fitness and LTL. Of course, longitudinal studies evaluating this are needed before definitive conclusions can be rendered. Future research, particularly studies employing objective measures of physical activity, is needed to better understand the mechanisms behind the observed associations. Several exercise-specific signaling mechanisms (e.g., TERT, IGF-1, eNOS, and AKT) may positively influence telomere biology by increasing telomere binding proteins and telomerase enzyme activity,^40,41^ and ultimately, preserve telomere phenotype.^41-43^

In conclusion, participants who engaged in more physical activity, sat less, and had higher cardiorespiratory fitness had the longest LTL. Notably, only MVPA was independently associated with LTL. Ultimately, these findings suggest that all three of the evaluated behavior characteristics, but particularly MVPA, may be important in preserving LTL. Thus, these findings underscore the importance of health professionals promoting MVPA-facilitating cardiorespiratory fitness as well as promoting strategies to minimize prolonged sedentary behavior. If future research confirms these findings, then this will have important implications for policy development among, for example, middle- and older-age adults within office-type work settings (e.g., designing work stations that minimize prolonged sedentary behavior).

## Ethical approval

This study was approved by the ethics committee at the National Center for Health Statistics. Consent was obtained from all participants prior to data collection.

## Competing interests

We declare no conflicts of interest.

## Authors’ contributions

All authors were involved in the conceptualization of the study, revising the manuscript and interpreting the results. Author MKE drafted the manuscript and author PDL computed the analyses.

## Acknowledgments

No funding was used to prepare this manuscript.

## References

[1] Blackburn EH (2000). Telomere states and cell fates. Nature.

[2] O’Sullivan RJ, Karlseder J (2010). Telomeres: protecting chromosomes against genome instability. Nat Rev Mol Cell Biol.

[3] Campisi J (2013). Aging, cellular senescence, and cancer. Annu Rev Physiol.

[4] Freund A, Orjalo AV, Desprez PY, Campisi J (2010). Inflammatory networks during cellular senescence: causes and consequences. Trends Mol Med.

[5] Chung HY, Cesari M, Anton S, Marzetti E, Giovannini S, Seo AY (2009). Molecular inflammation: underpinnings of aging and age-related diseases. Ageing Res Rev.

[6] Lopez-Otin C, Blasco MA, Partridge L, Serrano M, Kroemer G (2013). The hallmarks of aging. Cell.

[7] Huzen J, de Boer RA, van Veldhuisen DJ, van Gilst WH, van der Harst P (2010). The emerging role of telomere biology in cardiovascular disease. Front Biosci (Landmark Ed).

[8] Thompson PD, Buchner D, Pina IL, Balady GJ, Williams MA, Marcus BH (2003). Exercise and physical activity in the prevention and treatment of atherosclerotic cardiovascular disease: a statement from the Council on Clinical Cardiology (Subcommittee on Exercise, Rehabilitation, and Prevention) and the Council on Nutrition, Physical Activity, and Metabolism (Subcommittee on Physical Activity). Circulation.

[9] Loprinzi PD, Loenneke JP, Blackburn EH (2015). Movement-based behaviors and leukocyte telomere length among US adults. Med Sci Sports Exerc.

[10] Loprinzi PD (2015). Cardiorespiratory capacity and leukocyte telomere length among adults in the United States. Am J Epidemiol.

[11] Dunstan DW, Barr EL, Healy GN, Salmon J, Shaw JE, Balkau B (2010). Television viewing time and mortality: the Australian Diabetes, Obesity and Lifestyle Study (AusDiab). Circulation.

[12] Loprinzi PD (2015). Leisure-time screen-based sedentary behavior and leukocyte telomere length: implications for a new leisure-time screen-based sedentary behavior mechanism. Mayo Clin Proc.

[13] Bouchard C, Blair SN, Katzmarzyk PT (2015). Less sitting, more physical activity, or higher fitness?. Mayo Clin Proc.

[14] Loprinzi P (2015). Health-enhancing multibehavior and medical multimorbidity. Mayo Clin Proc.

[15] Loprinzi PD, Loenneke JP. Lower extremity muscular strength and leukocyte telomere length: implications of muscular strength in attenuating age-related chronic disease. J Phys Act Health. 2015. 10.1123/jpah.2015-0120 26314088

[16] Loprinzi PD, Sng E (2016). Mode-specific physical activity and leukocyte telomere length among U.S. adults: implications of running on cellular aging. Prev Med.

[17] Loprinzi PD, Ramulu PY (2013). Objectively measured physical activity and inflammatory markers among US adults with diabetes: implications for attenuating disease progression. Mayo Clin Proc.

[18] Cawthon RM (2002). Telomere measurement by quantitative PCR. Nucleic Acids Res.

[19] McDowell MA, Hughes JP, Borrud LG (2006). Health characteristics of U.S. adults by body mass index category: results from NHANES 1999-2002. Public Health Rep.

[20] Clark BK, Sugiyama T, Healy GN, Salmon J, Dunstan DW, Owen N (2009). Validity and reliability of measures of television viewing time and other non-occupational sedentary behaviour of adults: a review. Obes Rev.

[21] Loprinzi PD (2015). Dose-response association of moderate-to-vigorous physical activity with cardiovascular biomarkers and all-cause mortality: Considerations by individual sports, exercise and recreational physical activities. Prev Med.

[22] Loprinzi PD (2016). Multimorbidity, cognitive function, and physical activity. Age (Dordr).

[23] Ainsworth BE, Haskell WL, Whitt MC, Irwin ML, Swartz AM, Strath SJ (2000). Compendium of physical activities: an update of activity codes and MET intensities. Med Sci Sports Exerc.

[24] Du M, Prescott J, Kraft P, Han J, Giovannucci E, Hankinson SE (2012). Physical activity, sedentary behavior, and leukocyte telomere length in women. Am J Epidemiol.

[25] Jackson AS, Blair SN, Mahar MT, Wier LT, Ross RM, Stuteville JE (1990). Prediction of functional aerobic capacity without exercise testing. Med Sci Sports Exerc.

[26] American College of Sports Medicine. ACSM’s Guidelines for Exercise Testing and Prescription. 6th ed. Philadelphia, PA: Lippincott Williams and Wilkins Company; 1995.

[27] Shephard RJ, Allen C, Benade AJ, Davies CT, Di Prampero PE, Hedman R (1968). The maximum oxygen intake. An international reference standard of cardiorespiratory fitness. Bull World Health Organ.

[28] Noonan V, Dean E (2000). Submaximal exercise testing: clinical application and interpretation. Phys Ther.

[29] Bruce RA, Kusumi F, Hosmer D (1973). Maximal oxygen intake and nomographic assessment of functional aerobic impairment in cardiovascular disease. Am Heart J.

[30] Ebbeling CB, Ward A, Puleo EM, Widrick J, Rippe JM (1991). Development of a single-stage submaximal treadmill walking test. Med Sci Sports Exerc.

[31] Cooper KH (1968). A means of assessing maximal oxygen intake. Correlation between field and treadmill testing. JAMA.

[32] Brouilette SW, Moore JS, McMahon AD, Thompson JR, Ford I, Shepherd J (2007). Telomere length, risk of coronary heart disease, and statin treatment in the West of Scotland Primary Prevention Study: a nested case-control study. Lancet.

[33] Ye S, Shaffer JA, Kang MS, Harlapur M, Muntner P, Epel E (2013). Relation between leukocyte telomere length and incident coronary heart disease events (from the 1995 Canadian Nova Scotia Health Survey). Am J Cardiol.

[34] Biswas A, Oh PI, Faulkner GE, Bajaj RR, Silver MA, Mitchell MS (2015). Sedentary time and its association with risk for disease incidence, mortality, and hospitalization in adults: a systematic review and meta-analysis. Ann Intern Med.

[35] Pate RR, Pratt M, Blair SN, Haskell WL, Macera CA, Bouchard C (1995). Physical activity and public health. A recommendation from the Centers for Disease Control and Prevention and the American College of Sports Medicine. JAMA.

[36] Pate RR, O’Neill JR, Lobelo F (2008). The evolving definition of “sedentary”. Exerc Sport Sci Rev.

[37] Aires L, Silva P, Silva G, Santos MP, Ribeiro JC, Mota J (2010). Intensity of physical activity, cardiorespiratory fitness, and body mass index in youth. J Phys Act Health.

[38] Bouchard C, Daw EW, Rice T, Perusse L, Gagnon J, Province MA (1998). Familial resemblance for VO2max in the sedentary state: the HERITAGE family study. Med Sci Sports Exerc.

[39] Maes HH, Beunen GP, Vlietinck RF, Neale MC, Thomis M, Vanden Eynde B (1996). Inheritance of physical fitness in 10-yr-old twins and their parents. Med Sci Sports Exerc.

[40] Ludlow AT, Ludlow LW, Roth SM (2013). Do telomeres adapt to physiological stress? Exploring the effect of exercise on telomere length and telomere-related proteins. Biomed Res Int.

[41] Ludlow AT, Roth SM (2011). Physical activity and telomere biology: exploring the link with aging-related disease prevention. J Aging Res.

[42] Werner C, Furster T, Widmann T, Poss J, Roggia C, Hanhoun M (2009). Physical exercise prevents cellular senescence in circulating leukocytes and in the vessel wall. Circulation.

[43] Werner C, Hanhoun M, Widmann T, Kazakov A, Semenov A, Poss J (2008). Effects of physical exercise on myocardial telomere-regulating proteins, survival pathways, and apoptosis. J Am Coll Cardiol.

